# The first case series analysis on efficacy of esmolol injection for in-hospital cardiac arrest patients with refractory shockable rhythms in China

**DOI:** 10.3389/fphar.2022.930245

**Published:** 2022-09-30

**Authors:** Rui Lian, Guochao Zhang, Shengtao Yan, Lichao Sun, Wen Gao, Jianping Yang, Guonan Li, Rihong Huang, Xiaojie Wang, Renyang Liu, Guangqing Cao, Yong Wang, Guoqiang Zhang

**Affiliations:** ^1^ Emergency Department, China-Japan Friendship Hospital, Beijing, China; ^2^ General Surgery Department, China-Japan Friendship Hospital, Beijing, China; ^3^ Cardiac Care Unit, The First Affiliated Hospital of Dalian Medical University, Dalian, China; ^4^ Intensive Care Unit, Zhejiang Provincial People’s Hospital, Hangzhou, China; ^5^ Cardiac Surgery Department, Qilu Hospital of ShanDong University, Jinan, China; ^6^ Cardiac Care Unit, XiangTan Central Hospital, Xiangtan, China

**Keywords:** cardiac arrest, ventricular fibrillation, pulseless ventricular tachycardia, esmolol, return of spontaneous circulation (ROSC)

## Abstract

**Background:** This study assessed the effects of esmolol injection in patients with in-hospital cardiac arrest (IHCA) with refractory ventricular fibrillation (VF)/pulseless ventricular tachycardia (pVT).

**Methods:** From January 2018 to December 2021, 29 patients with IHCA with refractory shockable rhythm were retrospectively reviewed. Esmolol was administered after advanced cardiovascular life support (ACLS)-directed procedures, and outcomes were assessed.

**Results:** Among the 29 cases, the rates of sustained return of spontaneous circulation (ROSC), 24-h ROSC, and 72-h ROSC were 79%, 62%, and 59%, respectively. Of those patients, 59% ultimately survived to discharge. Four patients with cardiac insufficiency died. The duration from CA to esmolol infusion was significantly shorter for patients in the survival group (SG) than for patients in the dead group (DG) (12 min, IQR: 8.5–19.5 vs. 23.5 min, IQR: 14.4–27 min; *p* = 0.013). Of those patients, 76% (22 of 29) started esmolol administration after the second dose of amiodarone. No significant difference was observed in the survival rate between this group and groups administered an esmolol bolus simultaneously or before the second dose of amiodarone (43% vs. 64%, *p* = 0.403). Of those patients, 31% (9 of 29) were administered an esmolol bolus for defibrillation attempts ≤ 5, while the remaining 69% of patients received an esmolol injection after the fifth defibrillation attempt. No significant differences were observed in the rates of ≥ 24-h ROSC (67% vs. 60%, *p* = 0.73), ≥ 72-h ROSC (67% vs. 55%, *p* = 0.56), and survival to hospital discharge (67% vs. 55%, *p* = 0.56) between the groups administered an esmolol bolus for defibrillation attempts ≤ 5 and defibrillation attempts > 5.

**Conclusion:** IHCA patients with refractory shockable rhythms receiving esmolol bolus exhibited a high chance of sustained ROSC and survival to hospital discharge. Patients with end-stage heart failure tended to have attenuated benefits from beta-blockers. Further large-scale, prospective studies are necessary to determine the effects of esmolol in patients with IHCA with refractory shockable rhythms.

## 1 Introduction

Despite extensive research on cardiac arrest (CA), the morbidity and mortality of in-hospital cardiac arrest (IHCA) remain high. Ventricular fibrillation (VF)/pulseless ventricular tachycardia (pVT) is estimated to be the initial rhythm in approximately 15.6% of cases of sudden death in China (Zeng et al., 2013; Shao et al., 2016). The current consensus is that short intervals from collapse to the first defibrillation attempt may significantly improve survival after CA due to VF/pVT (Valenzuela et al., 2000). However, VF/pVT that remains refractory to the first few delivered shocks is associated with a poorer prognosis (Herlitz et al., 1997a). Indeed, the mortality of refractory shockable rhythms is between 86% and 97% (Herlitz et al., 1997b; Allegra et al., 2001; Dorian et al., 2002).

In addition to high-quality cardiopulmonary resuscitation (CPR) and early defibrillation, current ACLS algorithms for the management of VF/pVT recommend the sequential administration of epinephrine. Epinephrine predominantly exerts its effects *via* α-adrenergic signaling to improve coronary perfusion pressure (CPP), which is associated with an increased incidence of return of spontaneous circulation (ROSC). Moreover, β-adrenergic properties are likely to reduce the threshold of fatal arrhythmia (Bourque et al., 2007; Gough and Nolan, 2018). Thus, the utility of epinephrine in patients with CA remains controversial.

Abundant evidence from animal studies indicates that blocking the beta effects of the high catecholamine concentrations provides significantly better outcomes during CA from refractory VF/pVT (Nademanee et al., 2000; Bourque et al., 2007; Jingjun et al., 2009; Bassiakou et al., 2009; Andersen and Granfeldt, 2018; Aves et al., 2020). Only a limited number of case reports (the largest number of cases of patients with CA was 15) (Driver et al., 2014; Lee et al., 2016) have reported the successful use of esmolol in OHCA patients with refractory shockable rhythms. Due to poor awareness among physicians using esmolol in patients with CA and the difficulty of including such patients, there has been a lack of clear application status of esmolol among in-hospital patients with refractory VF/pVT and a paucity of established standard protocols for managing these patients in China. Since 2018, our team has been investigating patients with CA with refractory shockable rhythms and has administered esmolol as a rescue therapy after the sequential administration of epinephrine and anti-arrhythmic agents. This study aimed to report on a retrospective series of 29 IHCA patients with refractory VF/pVT treated with esmolol as a supplementary agent at our institution and within the network of baseline investigation of patients with CA in China (BASIC, NCT03926325) between January 2018 and December 2021. Our findings may provide useful information for future clinical trials. To the best of our knowledge, this is the first case series analysis on the efficacy of esmolol administration for patients with IHCA with refractory shockable rhythms in China.

## 2 Patients and methods

### 2.1 Patient eligibility

The files of patients administered esmolol by intravenous injection for refractory VF/pVT between January 2018 and December 2021 were reviewed. We identified 29 CA patients from IHCA with refractory shockable rhythms defined as VF/pVT that persisted after at least three defibrillation attempts treated with esmolol at China-Japan Friendship Hospital, Beijing (10 patients), and all patients were included in the database of the BASIC network by other six institutions (The First Affiliated Hospital of Dalian Medical University, seven patients; Zhejiang Provincial People’s Hospital, three patients; The First Affiliated Hospital of Harbin Medical University, two patients; XiangTan Central Hospital, two patients; Qilu Hospital of Shandong University, four patients; The First Hospital of Lanzhou University, one patient). As all patients with COVID-19 in China are admitted to designated infectious disease hospitals or mobile cabin hospitals, which were not included in the database of the BASIC network, no patients with COVID-19 were included in this study. Patients with CA with refractory VF/pVT were included based on the following criteria: 1) patients aged ≥18 years; 2) detailed medical records were available, including records of ACLS procedures, electrocardiogram, lab tests, and radiological images; 3) the initial rhythm was VF or VT refractory to at least three defibrillation attempts, including defibrillation after administration of standard ACLS medications; 4) received esmolol during CA. Exclusion criteria were 1) esmolol was not administered during the period of CA (including those who received esmolol before or after CA); 2) medical records were insufficient to analyze treatment outcomes; 3) recent medication history of beta-blockers or anti-arrhythmic medications. Informed consent forms for the use of clinical data for medical research were signed by each patient or their family members. The study protocol was approved by the local ethics committee.

### 2.2 Clinical assessments

**FIGURE 1:**
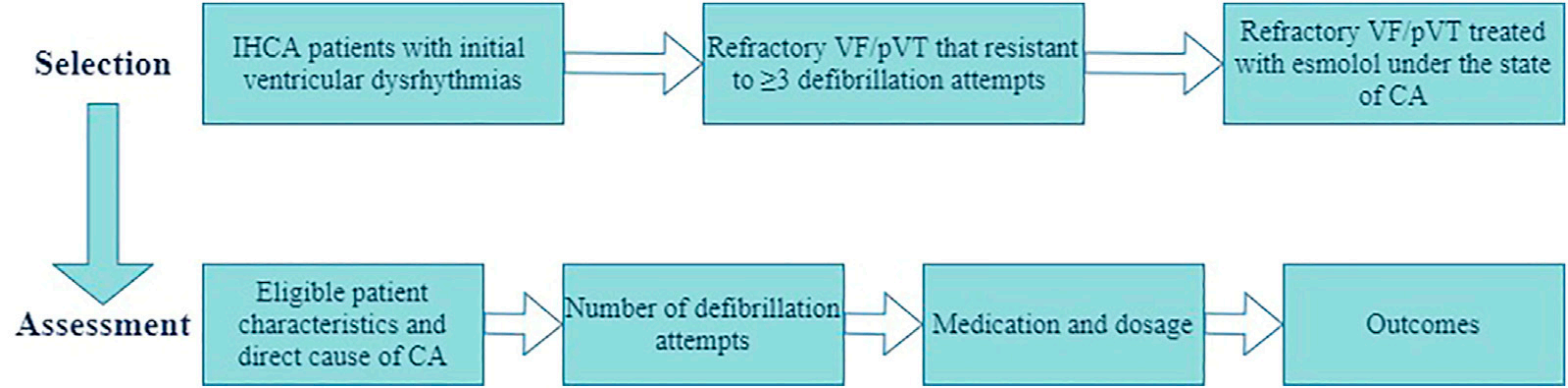
Patient selection and assessment fluxogram.

The patient selection and assessment are presented in Figure 1. Data collected from patient records included patient characteristics and direct cause of CA, reported epinephrine and anti-arrhythmic drug use, number of defibrillations, treatment modalities, duration of resuscitation, and outcomes. Response to treatment was assessed with the criteria for evaluating ROSC based on the 2015 American Heart Association guidelines. Efficacy assessment included sustained ROSC (≥20 min of spontaneous circulation without recurrence of CA), ≥24-h ROSC, ≥72-h ROSC, and survival to hospital discharge.

### 2.3 Treatment

Patients received esmolol after obtaining verbal informed consent from their proxies during the resuscitative effort. Written informed consent was obtained after the resuscitation. Esmolol was initially bolus injected intravenously to achieve a loading dose, followed by continuous infusion.

### 2.4 Statistical analysis

Descriptive statistics were used to summarize patient characteristics. Values are expressed as medians and interquartile ranges (IQRs). Intergroup differences were evaluated using the independent two-sample *t* test, chi-square test, or Fisher’s exact test. All *p* values that are equal to 0.05 or less were considered statistically significant. Analyses were performed using SPSS software (version 22.0; SPSS Inc., Chicago, IL, United States).

## 3 Results

### 3.1 Patients

**FIGURE 2:**
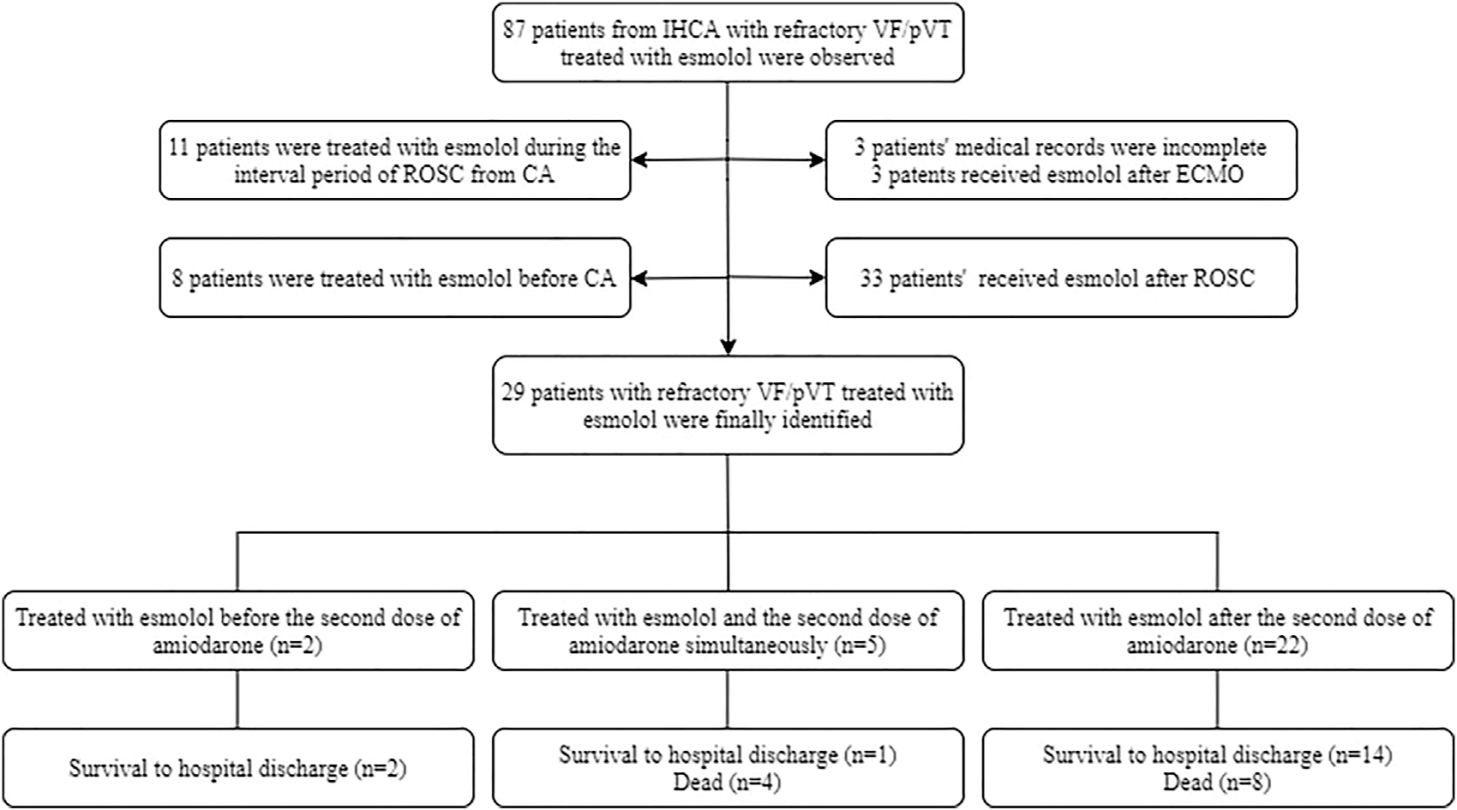
Patient flowchart and brief results.

**FIGURE 3:**
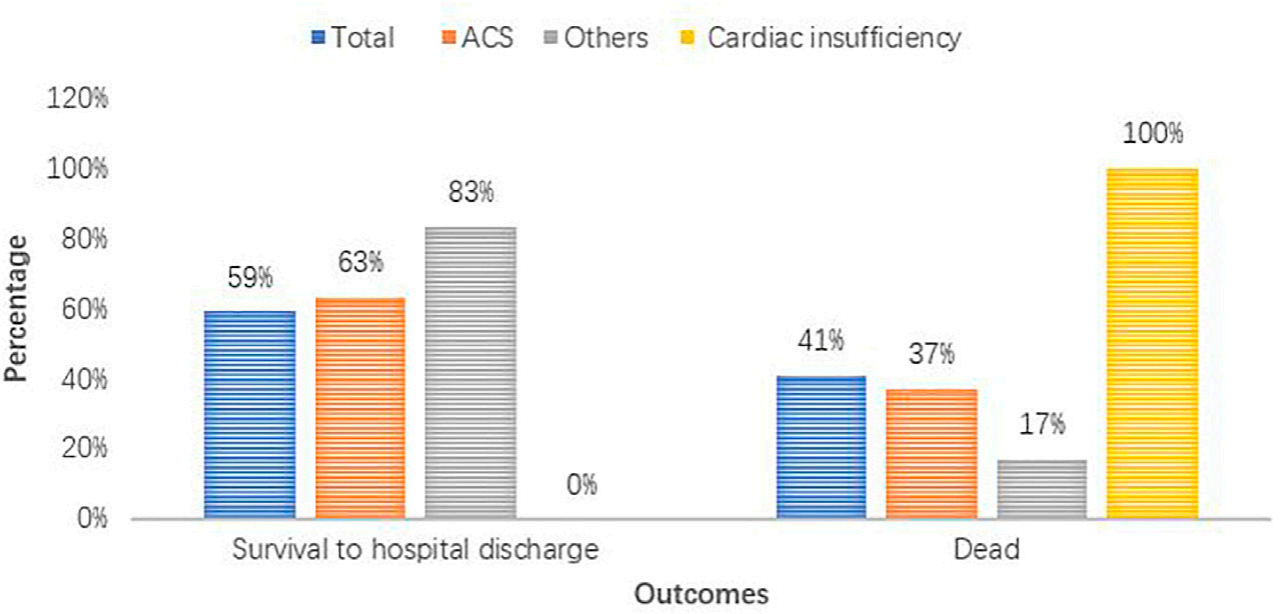
Patient outcomes for different diagnoses.

At the start of the study, a total of 87 patients from IHCA with refractory shockable rhythms treated with esmolol were observed. Of those patients, three opted to use esmolol after establishing extracorporeal membrane oxygenation (ECMO). Three patients had incomplete medical records, and 11 patients were treated with esmolol during the interval period of ROSC from CA. Eight patients selected esmolol therapy before CA, and 33 patients received esmolol after ROSC. In total, 29 patients with persistent or recurrent VF/pVT received esmolol treatment in the real absence of spontaneous circulation conditions, comprising 25 patients with persistent VF/pVT during CPR and four patients with recurrence after standard ACLS. Therefore, these 29 cases were included in the final analysis. The patient flowchart and enrolment are presented in Figure 2. Of the 29 cases, 15 (52%), 6 (21%), 3 (14%), 4 (10%), and 1 (3%) were reported from the emergency room (ER), cardiac intensive care unit (CCU), cardiology, intensive care unit (ICU), and operation room (OR), respectively (Supplementary Figure S1). At baseline, all 29 patients who were not previously treated with beta-blockers experienced IHCA and initially received standard manual CPR by first responders. Of these patients, 19 were diagnosed with acute coronary syndrome (ACS), four with cardiac insufficiency (two with ischemic cardiomyopathy and two with dilated cardiomyopathy), three had complications after valve replacement, two had cardiac ion channel disease, and one had severe electrolyte disturbance. Patient outcomes for different diagnoses are shown in Figure 3. The median age of patients who received esmolol during CPR was 65 years (IQR: 52–74 years), with an unequal sex distribution (79.3% were men and 20.7% were women). The median number of defibrillation attempts during CPR was nine (IQR: 7–12) times. The median number of defibrillations before and after esmolol administration was six (IQR: 5–7.5) and four (IQR: 1.5–5) times, respectively. The median time between CA and the first dose of traditional anti-arrhythmic agents was 6 (IQR: 5–8) min. The median time between CA and initiation of esmolol bolus was 15 min (IQR: 10.5–23.5 min). The median doses of epinephrine, amiodarone, and esmolol were 13 (IQR: 8.5–19.5) mg, 450 (IQR: 300–450) mg, and 60 (IQR: 53.5–70) mg, respectively. Patient characteristics are listed in Table 1. All patients had documented VF/pVT as their initial rhythm before starting esmolol treatment.

**TABLE 1:** Baseline demographic and clinical characteristics of IHCA patients with esmolol (N = 29).

Characteristics	IHCA patients with esmolol (*n* = 29)
Age, median (range), years	65 (52–74)
Male, n (%)	23 (79%)
Primary diagnosis, n (%)	
ACS	19 (66%)
Cardiac insufficiency	4 (14%)
Fulminant myocarditis	1 (3%)
Complications after surgery	2 (7%)
Cardiac ion channel disease	2 (7%)
Disturbance of electrolyte	1 (3%)
Initial rhythm, n (%)	
VF	25 (86.2%)
pVT	4 (13.8%)
Number of defibrillation attempts, median (IQR)	
Median number during CPR	9 (7–12)
Median number before esmolol administration	6 (5–7.5)
Median number after esmolol administration	4 (1.5–5)
Time point of drug use (min)^a^, median (IQR)	
Amiodarone use	6 (5–8)
Esmolol use	15 (10.5–23.5)
Dosage(mg), median (IQR)	
Epinephrine	13 (8.5–19.5)
Amiodarone	450 (300–450)
Esmolol	60 (53.5–70)
Duration of CPR (min), median (IQR)	42 (25.5–55.5)
Outcomes, n (%)	
Sustained ROSC	23 (79.3%)
≥24-h ROSC	18 (62.1%)
≥72-h ROSC	17 (58.6%)
Survival to hospital discharge	17 (58.6%)

a Time point of drug use: from the time of CA to drug administration.

### 3.2 Treatment

Most patients had received recommended doses of adrenaline and anti-arrhythmic agents according to the ACLS protocol but still had a rhythm of VF/pVT at the time of esmolol administration. Of those patients, seven (24%) were administered an esmolol bolus concurrently or before the second dose of amiodarone, while the remaining 22 (76%) were administered esmolol after the second dose of amiodarone. All patients received an esmolol loading dose and infusion at 500–1000 and 0–100 mcg/kg/min, respectively. The 29 patients were divided into a survival group (SG) and a dead group (DG) for further comparison (Table 2). Age, sex, anti-arrhythmic drug dose, and number of defibrillation attempts were similar between groups (Table 2). CPR to esmolol duration was significantly shorter in the SG than in the DG (12 min, IQR: 8.5–19.5 vs. 23.5 min, IQR: 14.4–27 min; *p* = 0.013). No difference was observed in amiodarone and esmolol doses between SG and DG. In contrast, the DG received higher dosages of total epinephrine (18 mg, IQR: 10.3–24.5 mg) and epinephrine before esmolol administration (9 mg, IQR: 7–13.75 mg) than the SG (total epinephrine was 11 mg, IQR: 7–17 mg; epinephrine before esmolol administration was 5 mg, IQR: 2.5–8.5 mg) throughout CPR (*p* < 0.05).

**TABLE 2:** Comparison of baseline characteristics and interventions between SG and DG.

	SG (*n* = 17)	DG (*n* = 12)	*p*-value
Male, n (%)	14 (82)	9 (75)	0.669
Age, median (range), and years	60 (49.5–70.5)	67.5 (57.3–74.8)	0.315
Time point of drug use (min)			
Time point of amiodarone use^a^	5 (4–7)	7 (6–8)	0.114
Time point of esmolol use^b^	12 (8.5–19.5)	23.5 (14.4–27)	0.013
Dosage			
Total epinephrine	11 (7–17)	18 (10.3–24.5)	0.043
Epinephrin before esmolol	5 (2.5–8.5)	9 (7–13.75)	0.042
Epinephrin after esmolol	8 (4.5–13.5)	3.5 (2.25–7.75)	0.106
Amiodarone	450 (300–450)	450 (300–450)	0.905
Esmolol	60 (57–70)	62.5 (50–75.3)	0.869
Defibrillation attempts			
Median number during CPR	11 (7–12)	7.5 (7–12)	0.285
Median number before esmolol administration	6 (4.5–7)	6 (5.3–8.8)	0.346
Median number after esmolol administration	4 (2–6)	2 (1–4)	0.117

a Time point of amiodarone use: from the time of CA to amiodarone administration.

b Time point of esmolol use: from the time of CA to esmolol administration.

### 3.3 Efficacy of esmolol

In the 29 esmolol-treated patients, ≥72-h ROSC and survival to hospital discharge were both achieved in 17 cases (59%). Of those 29 cases, 18 (62%) exhibited ≥24-h ROSC. The majority of patients achieved at least 20 min ROSC (79%). The overall survival rate was 59%.

Of the 23 patients with sustained ROSC, the median interval from the start of esmolol bolus to the observation of ROSC was 15 (IQR: 10.5–23.5) min. All four patients with cardiac insufficiency ultimately died (100%). The corresponding numbers of patients diagnosed with ACS and others were 7 (37%) and 1 (17%), respectively. The proportion of acute coronary syndrome was higher in the SG (76% vs. 58%, *p* = 0.422). The characteristics of six patients who did not achieve sustained ROSC are presented in Supplementary Table S1. Of the six patients, three (50%) were finally diagnosed with ST-segment elevation myocardial infarction (STEMI), while the other three (50%) were diagnosed with cardiac insufficiency. Of those patients with cardiac insufficiency, 75% failed to achieve sustained ROSC during the first 20 min of resuscitation.

**FIGURE 4:**
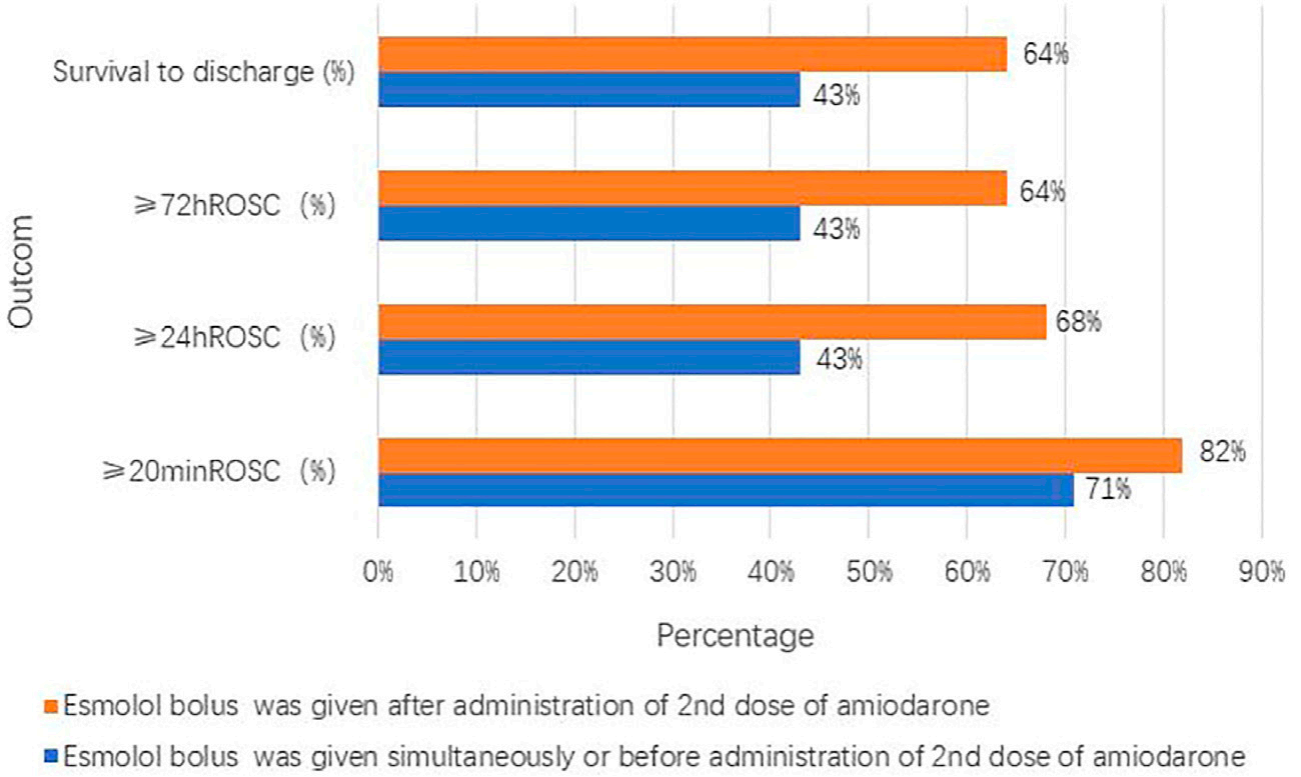
Groups with different timings of esmolol administration compared with the timing of amiodarone administration.

**FIGURE 5:**
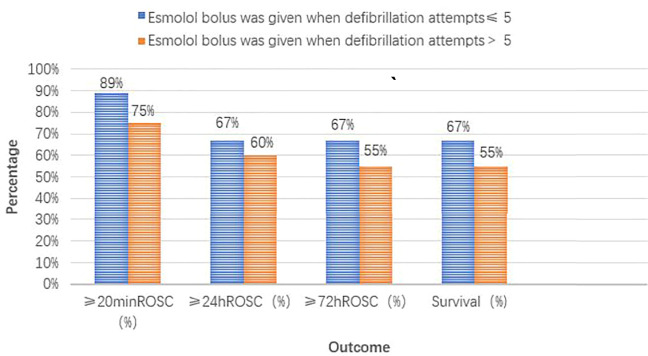
Different timing groups of esmolol administration (compared with defibrillation attempt timing).

The groups with different timings of esmolol administration are presented in Figures 4 and 5. Of the seven patients who started esmolol administration concurrently or before the second dose of amiodarone, three (43%) achieved 72-h ROSC and survival to hospital discharge, two (29%) were diagnosed with cardiac insufficiency caused by dilated cardiomyopathy, and two were (29%) diagnosed with acute extensive anterior STEMI and eventually died. Of the 22 patients who started esmolol administration after the second dose of amiodarone, 14 (64%) achieved 72-h ROSC and survival to hospital discharge, and eight (36%) eventually died. Among the eight patients who died, five (63%), two (25%), and one (12%) suffered from large myocardial infarction, cardiac insufficiency, and postsurgical complications, respectively. No significant difference was observed in the survival rate between the two groups (43% vs. 64%, *p* = 0.403). Of those 29 patients, nine were administered an esmolol bolus when defibrillation attempts ≤ 5, while the remaining 20 received an esmolol injection after the fifth defibrillation attempt. In the group administered an esmolol bolus when defibrillation attempts ≤ 5, one patient died within 20 min, and three other patients died within 24 h. Of the nine patients in the group administered an esmolol bolus given when defibrillation attempts≤ 5, the median duration from CA to esmolol infusion was 8 (range: 6–24) min. Of the 20 patients administered an esmolol bolus after the fifth defibrillation attempt, 15 had sustained ROSC, 12 had ≥24-h ROSC, and 11 survived to discharge. The median interval from CA to esmolol infusion was significantly shorter for the group with defibrillation attempts ≤ 5 than for these 20 patients (*p* = 0.011). Patients who received an esmolol bolus when defibrillation attempts ≤ 5 exhibited a trend for higher rates of ≥ 24-h ROSC, ≥ 72-h ROSC, and survival to hospital discharge compared to the defibrillation attempts ≥ 5 group (67% vs. 60%, 67% vs. 55%, and 67% vs. 55%, respectively) (Figure 5), but these differences did not reach statistical significance due to the small sample size.

## 4 Discussion

There is currently a lack of effective treatments for refractory VF/pVT, which is considered a critical status (Shih et al., 2007; Sakai et al., 2010; Reynolds et al., 2013; Soar et al., 2019). Our study demonstrated that administration of an esmolol bolus to patients with IHCA with refractory shockable rhythms may result in good clinical outcomes.

### 4.1 Medicinal agents in advanced cardiovascular life support

#### 4.1.1 Epinephrine

The PARAMEDIC-2 study results (Perkins et al., 2018) resulted in the emergence of controversy regarding the benefits of epinephrine for CA patients with both shockable and non-shockable initial rhythms (Tang et al., 1995; Niemann James and Garner, 2005; Bourque et al., 2007; Eifling et al., 2011; Gao and Sapp, 2013; Gough and Nolan, 2018; Jasmeet, 2020). The possibility of a refractory shockable rhythm depends predominantly on excessive endogenous catecholamines, sympathetic activation, and deleterious effects of β-adrenoceptor stimulation (Tang et al., 1995; Eifling et al., 2011; Gao and Sapp, 2013). In this case series of refractory VF/pVT, we observed that total epinephrine dosage and dosage of epinephrine before esmolol administration were significantly higher in the DG (*p* < 0.05). These data highlight the difficulties of managing individuals with refractory VF/pVT arrest due to a poor response to conventional resuscitation maneuvers including cumulated dosage of epinephrine. Therefore, aggressive therapies for CA are urgently needed.

#### 4.1.2 Anti-arrhythmic agents

Current guidelines recommend intravenous anti-arrhythmic agents (amiodarone and lidocaine) for the management of VF/pVT. However, our study demonstrated that 22 of 29 patients with CA (76%) did not respond to the second dose of amiodarone (Figure 1). Moreover, both the ARREST (Kudenchu et al., 1999) and ALIVE trials (Dorian et al., 2002) failed to confirm the effects of these agents on long-term survival. In these circumstances, clinicians often face challenges due to limited evidence-based treatment options.

#### 4.1.3 Other interventions

Several studies (Hassan et al., 2002; Harayama et al., 2014) have suggested new medicinal agents as an adjunct to existing ACLS procedures. However, none of these proposed drugs such as magnesium sulfate and nifekalant is sufficient to reverse a “shock-resistant” critical state. Notably, using extracorporeal CPR (ECPR) has recently been reported as a promising intervention in patients with refractory VF/pVT (Siao et al., 2015; Stub et al., 2015). However, ECPR is not routinely available and is restricted due to cost, complications, local medical conditions, and patients’ characteristics.

### 4.2 β-blockade administration

Since the mid-1990s, studies (Ditchey et al., 1992; Ditchey and Slinker, 1994; Killingsworth et al., 2004; Bassiakou et al., 2008) have reported that the administration of propranolol/esmolol/atenolol during CPR significantly decreased the fibrillating myocardium contraction force, thus reducing myocardial oxygen requirements. Moreover, several animal studies (Theochari et al., 2008; Jingjun et al., 2009; de Oliveira et al., 2012; Laura et al., 2021) have confirmed that higher rates of temporary ROSC, sustained ROSC, and neuroprotection were achieved using β-blockade. However, few studies (Driver et al., 2014; Lee et al., 2016) focusing on the use of esmolol in real clinical scenarios are available. In addition, concerns (Laurent et al., 2002; Hwang et al., 2019) regarding potential adverse negative inotropic effects associated with β-blockers on post-resuscitation myocardial function have emerged. In relation to these deleterious effects, Strohmenger et al. (1999) reported that myocardial function indices were lower in the epinephrine group than in the placebo group only in the first 5 min after ROSC. The distinguishing feature of esmolol is its rapid onset and short duration of action, which allows for a rapid return to pre-arrest cardiac function. Thus, most clinical cases (Nademanee et al., 2000; Driver et al., 2014; Lee et al., 2016; Gottlieb et al., 2019; Manogaran and Yang, 2020) have attempted to use esmolol with a 300–500 mcg/kg loading dose after the failure of standard CPR.

Despite the rarity of such situations (Gottlieb et al., 2019; Manogaran and Yang, 2020) in real-world settings, we retrospectively collected data on 29 patients with IHCA treated with esmolol. In our study, 74% (17 of 23) of patients with sustained ROSC survived long-term, which is consistent with the rate of 75% (3 of 4) reported by Driver et al. (2014) but was higher than the findings of Lee et al. (2016) (3 of 9; 33%). This difference may be related to the occurrence location of CA, absence or presence of witness by the first responder, and quality of basic CPR, since cases in our study were all witnessed IHCA cases provided from large tertiary hospitals. Overall, we observed a 59% survival to discharge rate, which was higher than the reported average survival (Sakai et al., 2010). Moreover, over two-thirds of patients had experienced more than five defibrillation attempts before receiving an esmolol bolus in our study. This indicates that esmolol was used as definite salvage therapy in our study. With regard to the significantly higher number of defibrillation attempts in our study, the outcome of higher survival rates is promising.

### 4.3 Administration time of β-blockade

Despite the small sample size of both groups, the interval from CA to esmolol infusion was shorter in the SG than in the DG. This may be related to the higher number of defibrillation attempts, resulting in temporary or permanent myocardial damage (Walcott et al., 2003; Lin et al., 2006). These data indicate that the optimal time of esmolol bolus may be appropriately shifted to an earlier timepoint in cases of poor prognosis caused by prolonged cardioplegia state and ischemia/hypoxia injury. Moreover, no difference was identified in sustained ROSC and survival rate between the group administered an esmolol bolus simultaneously or before the second dose of amiodarone and the group administered an esmolol bolus after the second dose of amiodarone. This indicates that the failure to respond to traditional anti-arrhythmia agents prior to esmolol bolus administration did not preclude sustained ROSC or survival to discharge.

### 4.4 Etiologies of in-hospital cardiac arrest with refractory shockable rhythms

The final diagnosis of 29 cases needs to be highlighted. ACS was the most common etiology in our study, accounting for 66% of cases. This result is in accordance with the study of 5,516 sudden death autopsy cases in China recently reported by Zhao et al. (2020). Among our cases, more than 70% of the etiologies could be attributed to ACS and cardiac ion channel disease. A potential explanation is that shock-resistant rhythms occur most often in individuals with coronary artery disease and inherited arrhythmic syndromes (Polentini et al., 2006; Peichl et al., 2021). Of the four cardiac insufficiency cases, two (50%) failed to achieve sustained ROSC and the other two did not survive to discharge, suggesting that end-stage heart failure is associated with poorer clinical outcomes and may not be a reasonable indication for beta-blocker use (Park et al., 2022). These results highlight candidates that would gain the most benefit from esmolol administration during CA.

### 4.5 Limitations

This study has several limitations. It was a retrospective observation, and the population sample was small. Furthermore, clinicians in China may hesitate to take the risk of using beta-blockers in patients with a complete absence of spontaneous circulation. Selection bias and unduly favorable outcomes may have occurred due to non-consecutive enrollment. Moreover, the optimal esmolol administration timepoint and precise patient population that would benefit remain inconclusive.

## 5 Conclusion

Refractory VF/pVT, resistant to conventional CPR, is associated with a high mortality rate. In our observation of 29 IHCA patients with refractory VF/pVT who received esmolol, the success rates of sustained ROSC, 24 h ROSC, 72 h ROSC, and survival to hospital discharge were 79%, 62%, 59%, and 59%, respectively. However, there were no significant differences in the rates of ≥24-h ROSC, ≥72-h ROSC, and survival to hospital discharge between the group administered an esmolol bolus when defibrillation attempts ≤ 5 and the group with defibrillation attempts > 5. Patients with end-stage heart failure tended to have attenuated benefits from beta-blockers. Further prospective studies of β-blockade in patients with CA are warranted.

## Data Availability

The raw data supporting the conclusion of this article will be made available by the authors, without undue reservation.
