# Supplementary material for: The first case series analysis on efficacy of esmolol injection for in-hospital cardiac arrest patients with refractory shockable rhythms in China
Source: Front Pharmacol. 2022 Sep 30;13:930245. doi: 10.3389/fphar.2022.930245 (PMC9561246; doi:10.3389/fphar.2022.930245)
Supplement: Supplementary file 1 [file Table1.DOCX]

| **Patient ID** | **Age**  **(years)** | **Gender** | **Initial rhythm** | **Duration of CPR (min)** | **Numbers of defibrillation attempts** | **Defibrillation** **numbers before esmolol** | **Defibrillation** **numbers after esmolol** | **Time point of esmolol use(min)** | **Diagnosis** | |
| --- | --- | --- | --- | --- | --- | --- | --- | --- | --- | --- |
| 1 | 53 | Male | VF | 68 | 7 | 6 | 1 | 24 | Acute inferior and anterior  STEMI | |
| 2 | 76 | Female | VF | 48 | 7 | 6 | 1 | 12 | | Acute extensive anterior  STEMI |
| 3 | 61 | Male | VF | 56 | 8 | 6 | 2 | 14 | | Acute extensive anterior  STEMI |
| 4 | 33 | Male | VF | 33 | 12 | 6 | 6 | 28 | | Ischemic cardiomyopathy, Cardiac insufficiency |
| 5 | 64 | Male | VF | 46 | 13 | 9 | 4 | 32 | | Dilated cardiomyopathy, Cardiac insufficiency |
| 6 | 75 | Male | VF | 43 | 6 | 5 | 1 | 24 | | Dilated cardiomyopathy, Cardiac insufficiency |

**Table S1:** Characteristics of 6 patients who failed to achieve sustained ROSC

**Figure S1**: Distribution of surveyed IHCA patients with esmolol (N=29)
